# Supplementary material for: Analysis of a meningococcal meningitis outbreak in Niger – potential effectiveness of reactive prophylaxis
Source: PLoS Negl Trop Dis. 2019 Mar 11;13(3):e0007077. doi: 10.1371/journal.pntd.0007077 (PMC6428357; doi:10.1371/journal.pntd.0007077)
Supplement: S1 Table — Household and village relative risk and proportion of cases that had a past contact, estimated in the non-epidemic and epidemic periods. (DOCX) [file pntd.0007077.s001.docx]

| **Metric** | **Household (non-epidemic)** | **Household (epidemic)** | **Village (non-epidemic)** | **Village (epidemic)** |
| --- | --- | --- | --- | --- |
| **Relative risk** | 5.01 (1.96, 10.38) | 3.43 (1.70, 6.15) | 1.01 (0.76, 1.33) | 4.80 (3.92, 5.93) |
| **Relative risk ratio** | 1 (reference) | 0.69 (0.25, 2.06)  p=0.48 | 1 (reference) | 4.75 (3.37, 6.73)  p=<0.001 |
| **% cases that had a past contact** | 4.3% (1.7%, 8.6%) | 5.6% (2.8%, 9.5%) | 35.6% (29.5%, 42.1%) | 74.5% (70.4%, 78.3%) |
| **Risk ratio** | 1 (reference) | 1.29 (0.49, 3.77)  p=0.62 | 1 (reference) | 2.09 (1.75, 2.54)  p<0.001 |

**S1 Table.** **Clustering metrics at the household and village level, in the non-epidemic and epidemic periods.**

Household and village relative risk and proportion of secondary cases, estimated in the non-epidemic and epidemic periods.
